# Depth-dependent valence stratification driven by oxygen redox in lithium-rich layered oxide

**DOI:** 10.1038/s41467-020-20198-w

**Published:** 2020-12-11

**Authors:** Jin Zhang, Qinchao Wang, Shaofeng Li, Zhisen Jiang, Sha Tan, Xuelong Wang, Kai Zhang, Qingxi Yuan, Sang-Jun Lee, Charles J. Titus, Kent D. Irwin, Dennis Nordlund, Jun-Sik Lee, Piero Pianetta, Xiqian Yu, Xianghui Xiao, Xiao-Qing Yang, Enyuan Hu, Yijin Liu

**Affiliations:** 1 Beijing Synchrotron Radiation Facility, Institute of High Energy Physics, Chinese Academy of Science, 100049 Beijing, China; 2 Stanford Synchrotron Radiation Lightsource, SLAC National Accelerator Laboratory, Menlo Park, CA 94025 USA; 3 University of Chinese Academy of Sciences, 100049 Beijing, China; 4 Chemistry Division, Brookhaven National Laboratory, Upton, NY 11973 USA; 5 Department of Physics, Stanford University, Stanford, CA 94305 USA; 6 Beijing Advanced Innovation Center for Materials Genome Engineering, Key Laboratory for Renewable Energy, Beijing Key Laboratory for New Energy Materials and Devices, Institute of Physics, Chinese Academy of Sciences, 100190 Beijing, China; 7 National Synchrotron Light Source II, Brookhaven National Laboratory, Upton, NY 11973 USA

**Keywords:** Batteries, Batteries, Batteries

## Abstract

Lithium-rich nickel-manganese-cobalt (LirNMC) layered material is a promising cathode for lithium-ion batteries thanks to its large energy density enabled by coexisting cation and anion redox activities. It however suffers from a voltage decay upon cycling, urging for an in-depth understanding of the particle-level structure and chemical complexity. In this work, we investigate the Li_1.2_Ni_0.13_Mn_0.54_Co_0.13_O_2_ particles morphologically, compositionally, and chemically in three-dimensions. While the composition is generally uniform throughout the particle, the charging induces a strong depth dependency in transition metal valence. Such a valence stratification phenomenon is attributed to the nature of oxygen redox which is very likely mostly associated with Mn. The depth-dependent chemistry could be modulated by the particles’ core-multi-shell morphology, suggesting a structural-chemical interplay. These findings highlight the possibility of introducing a chemical gradient to address the oxygen-loss-induced voltage fade in LirNMC layered materials.

## Abstract (web summary)

Lithium-rich layered material deserves in-depth understanding because it has large capacity enabled by both cation and anion activities. Here, authors apply 3D spectro-tomography with nano resolution to reveal the multi-layer morphology and depth-dependent transition metal valence distribution associated with oxygen redox.

## Introduction

Lithium-ion battery (LIB) is crucial for a world based on clean energy. Its importance has recently been recognized by the awarding of 2019 Nobel prize in chemistry to scientists working in this field^1^. Despite its great success, there is always strong motivation to further increase the energy density of LIB, especially when it comes to the application in large systems such as electric vehicles. To reach this goal, an effective approach is to increase the energy density of the cathode, a major component of the LIB. Conventional cathodes are solely based on transition metal (TM) cation redox, limiting the maximum capacity and energy density that can be possibly achieved. In this context, lithium-rich nickel–manganese–cobalt (LirNMC) layered material stands out as a high energy cathode by utilizing both cation and anion redox^2–7^. Typically, LirNMC can deliver capacity up to 280 mAh g^−1^. Unfortunately, this material has not been put into practical application because its voltage keeps decreasing during cycling (commonly referred to as “voltage fade” in the battery community). Many research efforts have been devoted to understanding the origin of such voltage fade^8–11^, mostly at the atomic or the electrode level. To bridge the gap in the length scale, it is highly desirable to understand the material at the particle level, which is termed the mesoscale.

Material properties at the mesoscale are important for material design. For example, mesoscale structural and chemical defects were found to play a very significant role in affecting the overall electrochemical performance of the battery^12,13^. Depth-dependent redox heterogeneity within the cathode particles has also been reported to affect the charging behavior^14^ and the thermal stability^15–17^. It can be expected that compositional, chemical, and morphological understanding of LirNMC material at the mesoscale will provide insight into the mechanism of voltage fade issue and suggest possible measures to address this challenge.

In this work, we employ nano-resolution spectro-tomography to study a typical LirNMC material- Li_1.2_Ni_0.13_Mn_0.54_Co_0.13_O_2_, spatially resolving the morphological, compositional, and chemical features in three-dimensions (3D) within the LirNMC particles^18–20^. We observed a multi-layer morphology, which is very likely correlated with the depth-dependent chemistry. We further reveal the chemical and spatial dependence of oxygen redox behavior in LirNMC material, providing an in-depth understanding of the voltage fade issue. These findings suggest that a depth-dependent compositional engineering strategy^21^ could be a viable path for solving the voltage fade problem in the LirNMC material.

## Results

**Fig. 1:**
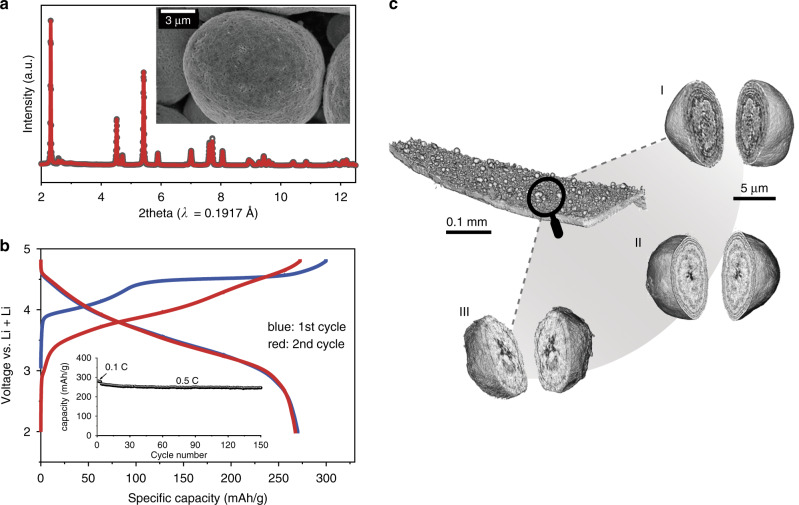
Electrochemical performance and morphology of LirNMC. **a** Structural characterization of LirNMC cathode material by XRD and Rietveld refinement. The inset graph shows the SEM image of LirNMC particles. **b** Charge-discharge profile and electrochemical cycling data of LirNMC | |Li metal half-cell. **c** Three-dimensional rendering of the composite electrode and magnified views of a few arbitrarily selected LirNMC cathodes particles (panels I, II, and III). The mesoscale core/multi-shell separation is clear visible in all the particles.

The composite battery cathode electrode is made of a large amount of several micron-sized active particles that are imbedded in a porous matrix of conductive carbon and binder domain (CBD). The active cathode particles are, ultimately, the energy reservoirs in the battery and their functionality relies on the CBD to provide mechanical support, electrical conductivity, and ion diffusion pathways. The synchrotron-based x-ray tomography technique has been demonstrated as a powerful tool for noninvasively reconstructing the 3D morphology of the cathode from the cell^22^ and the electrode^23^ scales down to the particle level^24–29^. As shown in Fig. 1a, the structure and morphology of the pristine material are characterized by x-ray diffraction (XRD) and scanning electron microscopy (SEM), respectively. Detailed results of the Rietveld refinement of LirNMC material are shown in Supplementary Table 1. XRD pattern indicates there is a honeycomb superstructure in the Li/TM layer as evidenced by the superlattice peaks on the right shoulder of the strongest XRD peak (lowering the space group symmetry from ${\mathrm{R}}\bar 3{\mathrm{m}}$ to C2/m). Recent studies by House and Bruce et al suggest that the honeycomb superstructure is closely related to the formation of O_2_ and oxygen vacancies^30^. The charge-discharge profile and the electrochemical cycling data are shown in Fig. 1b. The first cycle charge profile features a long plateau that is typical of the lithium-rich layered material and contributes to around two thirds of the whole charge capacity. As illustrated in Fig. 1c, we utilized the x-ray tomography to reconstruct the structure of a small piece of the LirNMC electrode. The studied electrode consists of a thin layer of active material and CBD (nearly a monolayer of LirNMC particles) on top of the aluminum current collector. High mass loading and close particle packing in a thick electrode (40–60 μm thick) is a common approach for improving energy density, which, however, can lead to significant particle-to-particle variation^23,31,32^. In our study, we choose to focus on the particle level because our scientific focus is the interplay between the particle’s morphology and the particle’s internal redox stratification. Therefore, a low mass loading is purposely chosen to minimize the cell polarization effect. More details about the electrode fabrication procedure can be found in the method section. To zoom in to the sub-particle level microstructures, we utilized x-ray nano-tomography to reconstruct the morphology of a few randomly selected LirNMC cathodes particles (2D projection images of several randomly selected particles are shown in Supplementary Fig. 1). As shown in Fig. 1c (panel I, II, and III), these LirNMC particles have multi-layer morphologies, distinguishing LirNMC from other cathode materials^33,34^. Although such morphology has been hinted previously in two-dimensional images^35^, our three-dimensional tomographic data offers unambiguous visualization and quantification, which we will demonstrate in detail later in this work. The multi-layer morphology may be due to intrinsic complexities of LirNMC. For example, LirNMC has multiple cations that differ greatly in size and reactivity. LirNMC also has structural complexity and the nature of its structure (whether a solid solution or a composite) has been debated for a long time^36–38^. The multi-layer configuration may have a profound impact on the redox chemistry within the particles as it adds more complexities to the lithium-ion diffusion and transition metal redox behavior within the particle.

**Fig. 2:**
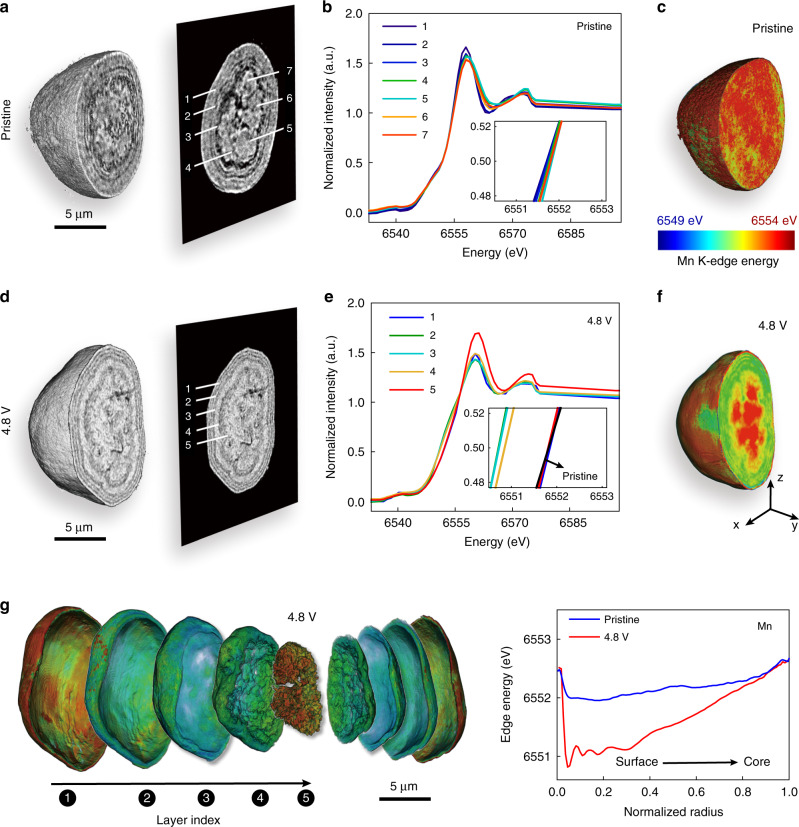
Spectro-microscopic investigation of the LirNMC particles. Panels (**a**) to (**c**) are on a pristine particle and panels (**d**) to (**g**) are on a charged particle (at 4.8 V in the first charge). Panels (**a**) and (**d**) are the 3D visualizations of the pristine and charged particles, with the labeling of different regions of interest on the respective central *xz*-slices. Panels (**b**) and (**e**) are the Mn K-edge x-ray absorption spectroscopic fingerprints over the regions of interest shown in panels (**a**) and (**c**), respectively. Panel (**c**) and (**f**) are the 3D renderings of the Mn’s valence state distribution in the pristine and the charged particles. Panel (**g**) is a detailed layer-by-layer rendering of the Mn’s valence state in the charged particle. Panel (**h**) is the comparison of the depth profiles of the particles in panels (**c**) and (**f**).

To gain the depth profile of transition metal valence, we turned to the spectro-tomography technique^39–42^ which could spatially resolve the local valence state of the element of interest in 3D. Such capability is achieved by conducting tomographic scans at a number of different energy levels across the absorption edge of the targeted element of interest. This technique is first applied to Mn, the most abundant transition metal in LirNMC. Two particles at the pristine and the charged (to 4.8 V in the initial cycle, at the fully charged state) states are scanned. As shown in Fig. 2a, d, the particle morphology is fairly similar and their Mn K-edge spectroscopic fingerprints over different regions (see labeling in the virtual slices through the center of the particle) are shown in Fig. 2b and **e**, respectively. The pristine particle appears to be relatively homogeneous in the Mn’s valence state as indicated by the depth-dependent Mn XANES plots in Fig. 2b and the 3D rendering of the Mn K-edge energy distribution in Fig. 2c. On the other hand, the charged particle clearly shows a depth-dependent Mn redox variation (see Fig. 2e, f, g). The core and the surface layer of the charged LirNMC particle exhibits high Mn valence state at 4^+^, while the Mn cations in the transition layers appear to be relatively reduced. These visual assessments of the pristine and the charged particles are further confirmed by the depth profile of Mn’s K-edge energy shown in Fig. 2h. While the relative homogeneity of the Mn valence state distribution in the pristine particle is anticipated^43^, the observed depth-dependent Mn valence in the charged LirNMC particle is somewhat a surprise, specifically because of its non-monotonicity. This observation motivates a more systematic study of all the transition metal cations (Mn, Co, and Ni) in the LirNMC particle in a correlative manner.

**Fig. 3:**
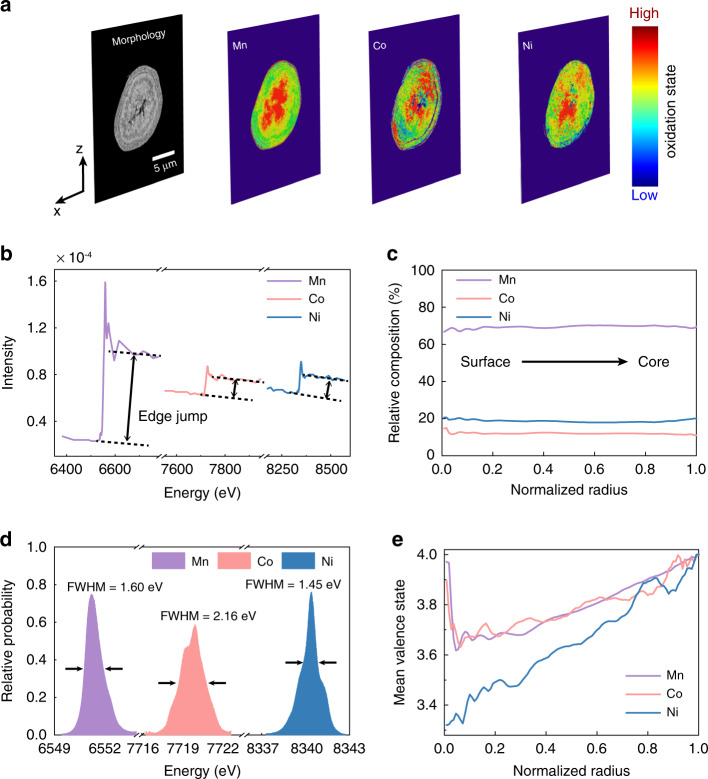
The spatial variation of the composition and the valence states of Mn, Co, and Ni in a charged (to 4.8 V) LirNMC particle. Panel (**a**) shows the valence states of Mn, Co, and Ni over the central xz slice of a charged LirNMC particle. Panel (**b**) illustrates the x-ray absorption spectroscopic fingerprints over a typical voxel in the reconstructed spectro-tomographic data. The edge jump is proportional to the relative elemental concentration and is utilized to quantify the elemental distribution in the particle volume. Panel (**c**) is the depth-dependent elemental concentration over the studied particle, suggesting the compositional homogeneity throughout the particle. Panel (**d**) is the relative probability distributions of the edge energy over the K-edges of Mn, Co, and Ni, respectively. Panel (**e**) shows the transition metal cations’ valence states variation over different depths.

**Fig. 4:**
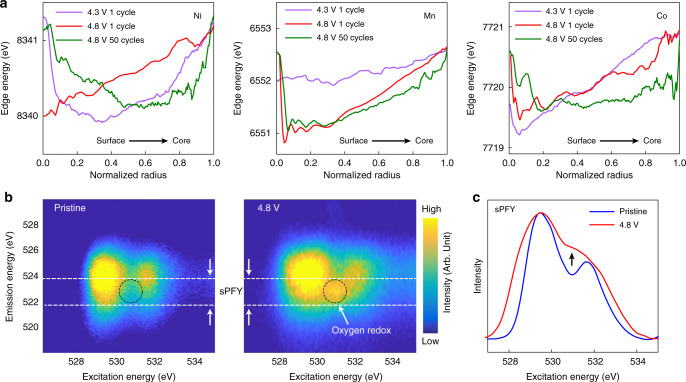
Depth-dependent redox behaviors in the LirNMC layered oxide cathode. The variations of the edge energies for all the three transition metal cations (Ni, Mn and Co) are shown in left, middle and right of panel (**a**), respectively. Soft x-ray RIXS data on the pristine and the charged (to 4.8 V in the first cycle) LirNMC samples are shown in panels (**b**). The comparison of the sPFY signals is shown in panel (**c**).

With the above-mentioned motivation, we carry out spectro-tomographic scans of a charged LirNMC particle over the absorption K-edges of the Mn, Co, and Ni, respectively, and correlatively. As shown in Fig. 3a, a depth-dependent redox heterogeneity can be observed in all the transition metal cations. To evaluate whether or not the compositional heterogeneity plays a role here, we first utilize the edge jump measurements on the K-edges of Mn, Co, and Ni (see Fig. 3b) to quantify the local elemental concentrations of these three metal elements^44^. The 3D elemental distributions are further quantified by evaluating their respective depth dependency (Fig. 3c), which suggests that the studied particle is relatively compositionally homogeneous throughout the particle volume. Correlating each pixel with edge position not only enables direct evaluation of 2D/3D images, but also provides a relative probability distribution as shown in Fig. 3d. The number of pixels featuring certain energy is divided by the number of total pixels to obtain the relative probability at that energy. Based on such probability distribution, we can further quantify the degrees of redox heterogeneity using the histogram plot of the 3D matrixes of edge energy. As shown in Supplementary Figs. 2–5, there are close relationship between edge position of the spectra and the valence of the transition metal. Therefore, the shape of the peak represents the relative probability distribution of the edge energy and the width of the peak effectively measures the degree of chemical nonuniformity. As shown in Fig. 3d, the Co’s valence state shows larger full width at half maximum (FWHM) in its histogram plot than that of the Mn and Ni. A quantitative evaluation of transition metal valence as a function of depth is shown in Fig. 3e. The valence of Ni is considerably low in the outermost layer and then gradually increases towards the core of the particle. The valences of Mn and Co are high in the outermost layer and then decrease going through the intermediate layer before increasing to high valence in the core area. In addition, in situ x-ray absorption spectroscopy (XAS) experiment at Ni, Mn, and Co K-edges was carried out (Supplementary Fig. 6) and the transition metal (mostly Ni) XAS spectra show obvious edge shift before the 4.5 V plateau and show no shift after the plateau begins. This suggests that as the oxidation of transition metal (mostly Ni) takes place way before the end of charge (transition metal oxidation contributes only around one third of the total capacity, followed by oxygen oxidation which contributes to two thirds), the low valence of transition metal is unlikely caused by insufficient oxidation, but by the oxygen redox around the transition metal cations. To confirm that the transition metal’s low valence is associated with oxygen redox, we further conduct spectro-tomography and compare the depth dependency of the redox profile (single pixel resolution) on particles at different states of charge (4.3 and 4.8 V in the first cycle) and after long-term battery operation (at 4.8 V after 50 cycles) shown in Fig. 4a for Ni, Mn, and Co, respectively. During the 1^st^ cycle charging (going from 4.3 to 4.8 V), the valence profiles of Ni and Mn change significantly and in very different ways while the profile of Co is relatively stable. At intermediate depths (excluding the surface vicinity as well as the center), the oxidation states increase for Ni but decrease for Mn. The Ni valence increase may be caused by incomplete Ni oxidation at 4.3 V and further charging leads to its valence increase. For Mn, the valence decreases going from 4.3 to 4.8 V. This may be because of the oxygen redox and the largest part of it could be associated with Mn. Detailed reasons will be explained later when discussing the valence profile change during cycling. The outcome of oxygen redox may be in the form of O^−^^45,46^, oxygen vacancy^9^, or O_2_ in the lattice^30^. They all can lead to a decrease in the valence of TM, or very likely Mn. For Co, it is well known that Co can form a very strong covalent bond with oxygen and this may contribute to the relatively unchanged valence profile of Co during charging. In the surface vicinity, or in the passivation layer, Ni and Mn can also behave differently. For example, NiMn_2_O_4_ is a commonly seen spinel phase arising from loss of lithium and oxygen in the layered material^9^. In NiMn_2_O_4_, Ni is in a low valence state (divalent) while Mn is in high valence state (between trivalent and tetravalent and can be higher if it is partially substituted by Li). In other words, the reconstructed surface may have low valence Ni but high valence Mn. Upon cycling, the valence profile evolves in different ways for Ni, Mn, and Co. The tetravalent Ni has been well known to have high oxygen partial pressure and oxygen around Ni can be easily released. This can lead to Ni valence decrease and may be related to the voltage fade issue. Our previous work suggested that continuous oxygen loss from the lattice is one of the main reasons for voltage fade in lithium-rich layered material. Similarly, the decrease of Co valence during cycling may also be linked with the voltage fade issue. For Mn, the valence profile shows relatively little change upon cycling. Interestingly, Gent et al showed that oxygen redox can be fairly reversible during cycling as indicated by the clearly visible oxygen oxidation RIXS feature in the long cycled sample^46^. These two observations may indicate that the largest part of reversible oxygen redox is coupled to Mn cations. Such argument is also supported by theoretical calculations showing that oxygen redox mainly takes place on those oxygen anions in the Li–O–Li moiety which has Li in the Li/TM layer^47^. The monovalent Li is most likely surrounded by high valent TM such as Mn (maybe also Co) because this helps to release the strain^48^. In other words, the active oxygen anions are most likely around the Mn cations.

In addition to the evolution of the redox heterogeneity, we conducted additional synchrotron nano-tomography on particles after 50 electrochemical cycles at a rate of 0.5 C and observed that clear cracks are populated in the particles after long-term cycling. It is useful to point out that the cracks are not confined in the particle core regions. Some of the cracks propagate through multiple shell layers, effectively open up the interior of the particle to the liquid electrolyte, forming fresh active solid-to-liquid interface that could facilitate the lithium exchange between the particle and the external environment (Supplementary Figure 7). The chemomechanical interplay in battery cathode particles is an active research field. Follow-up efforts for studying the particle morphological control as a viable method to mitigate the particle disintegration effect is highly desirable.

Valence heterogeneity is not a surprise as it has been observed in several cathode materials^22,27,33^. The unique TM valence profile in LirNMC is indicative of its own characteristic chemistry. In conventional layered NMC, the capacity is almost exclusively contributed by transition metal cations and oxygen anions do not participate in the redox reaction. For these materials (at charged state), the valence of Ni usually is highest in the outer layer and gradually decreases as the depth increases (disregarding the very surface layer of a few nanometers, which has other chemical complexities such as the surface reconstruction effect). This is suggesting a more complete TM oxidation in the outer layer where lithium ion can be more effectively extracted. However, in oxygen-active cathode material such as LirNMC, oxygen activity and the possible consequent oxygen vacancy induces an oxidation state depth profile that is significantly distinct from the conventional NMC.

Soft x-ray resonant inelastic x-ray scattering (RIXS), a powerful tool to study anion redox, is used in this study and the results are shown in Fig. 4b and **c**. At the charged state (Fig. 4b), a peak is clearly visible at excitation energy of 531 eV and emission energy of 523 eV, fingerprinting the formation of O^−1^ in the sub-surface transition layer of the material upon charging to high voltage^49–51^. The comparison of the super partial fluorescence yield (sPFY)^49^ signals from the pristine and the charged samples shown in Fig. 4c clearly support this observation. Althoughoxygen activity can increase the capacity and hence the energy density of cathode material, it would eventually lead to oxygen loss, leaving behind oxygen vacancy around the transition metal cations and decreasing their valences. Such uniqueness of the LirNMC explains why the TM valence has a very different profile from that of conventional NMC despite the fact that both have nonuniform lithium distribution in the charged particle.

## Discussion

To gain in-depth understanding of the voltage fade issue in LirNMC materials, synchrotron-based nano-resolution spectro-tomography is used to study the material Li_1.2_Ni_0.13_Mn_0.54_Co_0.13_O_2_ at the particle level, providing morphological, compositional, and chemical information. The unique multi-layer morphology of LirNMC is revealed in 3D with nanoscale resolution. The composition of the studied LirNMC is rather homogeneous throughout the pristine particle and not affected by charging. However, charging induces a depth-dependent profile of transition metal valence that is unique and distinguishes LirNMC from conventional NMC material. Such unique redox stratification is likely correlated with the depth-dependent association of oxygen redox with different transition metal cations. Our results highlight the importance of particle level engineering, which could be a key to maximize the effective electrochemical activity of the active materials.

## Methods

### Sample preparation

Li_1.2_Ni_0.13_Mn_0.54_Co_0.13_O_2_ compound was prepared by a conventional solid-state reaction using stoichiometric lithium carbonate (5% excess of lithium) and the metal hydroxide with Ni:Mn:Co of 0.13:0.54:0.13 in molar ratio. The mixture was firstly calcinated at 450 °C for 5 h, then calcinated at 850 °C for 12 h in air and cooled naturally. Electrodes were prepared by spreading the slurry (N-Methyl-2-pyrrolidone as the solvent) containing active materials (80 wt.%), acetylene carbon (10 wt.%) and polyvinylidene difluoride (PVDF, 10 wt.%) as the binder and casting it on carbon-coated aluminum foils. The electrodes were then dried overnight at 120 °C in a vacuum oven and transferred into an Ar-filled glovebox for future use. The active mass loadings for the electrodes were 1 mg cm^−2^. CR2032 coin cells were assembled in an Ar-filled glovebox (O_2_ < 0.5 ppm, H_2_O < 0.5 ppm) using the composite cathode, lithium foil (MTI) anode with thickness of 250 μm, Celgard 2500 as the separator and electrolyte made of 1 M LiPF_6_ dissolved in ethylene carbonate (EC) and ethyl methyl carbonate (EMC) (1:2 in volume). The LirNMC electrode was charged to 4.8 V using a Biological potentiostat/galvanostat (SP-300). The charging rate is C/8 corresponding to around 37.5 mA g^−1^. The cell was then disassembled in the Ar-filled glovebox and the electrode was thoroughly washed by dimethyl carbonate (DMC) solvents. The washed electrode was dried overnight in the Ar atmosphere before it is further characterized.

### Materials characterization

The crystal structure of the materials was characterized by powder x-ray diffraction (XPD) at 28-ID-2 beamline of the National Synchrotron Light Source II (NSLS-II) at Brookhaven National Laboratory (BNL) by a Perkin Elmer amorphous-Si flat panel detector, and the x-ray wavelength was 0.1917 Å. XRD refinement was conducted by the Rietveld method using the TOPAS^52^. The morphology of the materials was characterized by field emission scanning electron microscopy (FE-SEM, Hitachi 4800). The ex-situ and in situ x-ray absorption spectroscopy (XAS) experiments were performed in transmission mode using a Si (111) double-crystal monochromator at beamline 7-BM (QAS) of the NSLS-II at BNL. A reference spectrum for each element was simultaneously collected with the corresponding spectrum of the in-situ cells using transition metal foil. The energy calibration was carried out using the first inflection point of the K-edge spectrum of the transition metal foil as a reference. The XANES and extended x-ray absorption fine-structure data were analyzed using the ATHENA software package^53^.

### Nano-resolution spectro-tomography

X-ray micro-CT scan of a small piece of LirNMC cathode electrode was performed using Xradia Zeiss 520 Versa at Stanford Nano Shared Facilities (SNSF). In this scan, 1600 projections were collected from −90° to + 90°. More details of this equipment could be found elsewhere^36^.

The spectro-tomography was performed using the transmission x-ray microscopy (TXM) at the beamline 6-2c of Stanford Synchrotron Radiation Lightsource (SSRL) of SLAC National Accelerator Laboratory or at beamline 18-ID (FXI) of National Synchrotron Light Source II (NSLS II) at BNL. The nominal spatial resolution of TXM is ~30 nm. The LirNMC particles were first carefully peeled off from the Al current collector and then loaded in the quartz capillary (100 microns in diameter, 10 microns in wall thickness). During the 3D spectro-tomography scan, the energy scan across the absorption K-edges of Mn, Co, and Ni were carried out. The energy range of the incident x-ray is 6384 eV to 6776 eV for Mn, 7554 eV to 7946 eV for Co, and 8178 eV to 8570 eV for Ni, respectively. For each of these three edges, tomography was performed at 63 different energy points. In the near edge regions, (6534–6576 eV for Mn, 7704–7746 eV for Co, and 8328–8370 eV for Ni, respectively), the energy step was set to 1 eV to ensure sufficient energy resolution. The energy step size of the pre-edge and post-edge regions were set to 10 eV for covering a relatively wide energy window for normalization of the spectra. The 3D tomographic reconstruction and the XANES spectra analysis were carried out using an in-house developed software package known as TXM-Wizard^40^.

### Resonant inelastic x-ray scattering (RIXS)

Soft XAS and RIXS experiments were conducted at beamline 10-1 at Stanford Synchrotron Radiation Lightsource (SSRL)^54^. More experiment details about the soft x-ray spectroscopic measurements can be found in our previous work^5^.

## Supplementary information

Supplementary Information

## Data Availability

The data that support the findings within this paper are available from the corresponding authors on request.
