# Supplementary material for: Depth-dependent valence stratification driven by oxygen redox in lithium-rich layered oxide
Source: Nat Commun. 2020 Dec 11;11:6342. doi: 10.1038/s41467-020-20198-w (PMC7733467; doi:10.1038/s41467-020-20198-w)
Supplement: Supplementary file 1 — Supplementary Information [file 41467_2020_20198_MOESM1_ESM.pdf]

Supplementary information for

Depth-dependent valence stratification driven by oxygen redox in lithium-rich layered oxide

Zhang et al.

.

Supplementary Table

Supplementary Table 1 The refined structure of the pristine $Li_{1.2}Mn_{0.54}Co_{0.13}Ni_{0.13}O_2$ using the XRD data.

Space group: *C*2/m *a*=4.9357(4) Å, *b*=8.5525 (7) Å, *c*=5.0164(3) Å, *β*=108.96(6) °						
Atom	Wyckoff site	*x*	*y*	*z*	Occupancy	B_{iso} (Å^2)
Li	4h	0	0.306(3)	0.5	1	0.21(5)
Mn	4g	0	0.169(4)	0	0.81	0.68(6)
Co	4g	0	0.169(4)	0	0.19	0.68(6)
Li	2a	0	0	0.5	1	0.20 (3)
Li	2b	0	0.5	0	0.61	0.21 (3)
Ni	2b	0	0.5	0	0.39	0.21 (3)
O	4i	0.259(7)	0	0.207(6)	1	1.50(2)
O	8j	0.240(5)	0.340(2)	0.227(4)	1	0.26(1)

Supplementary Figures

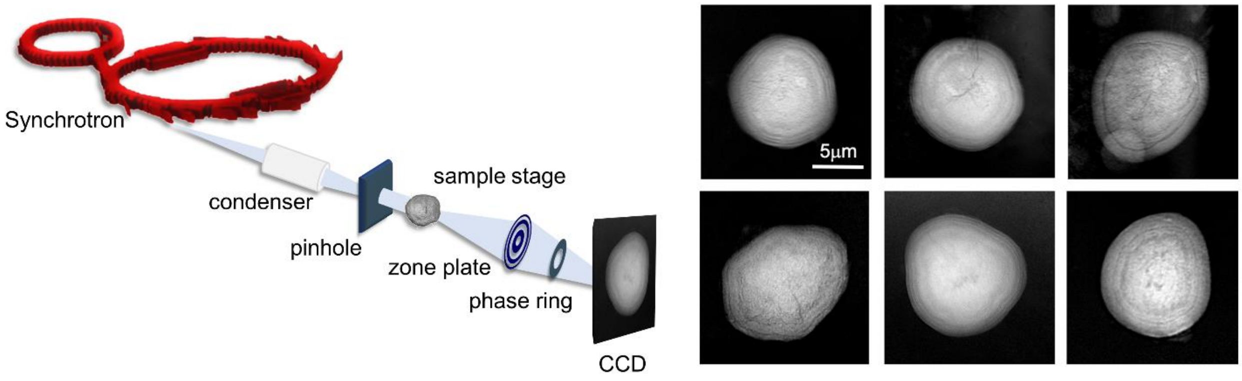

Supplementary Figure 1. A schematic illustration of synchrotron nano-tomography technique is shown on the left. Two-dimensional projection images of several randomly selected particles are shown on the right.

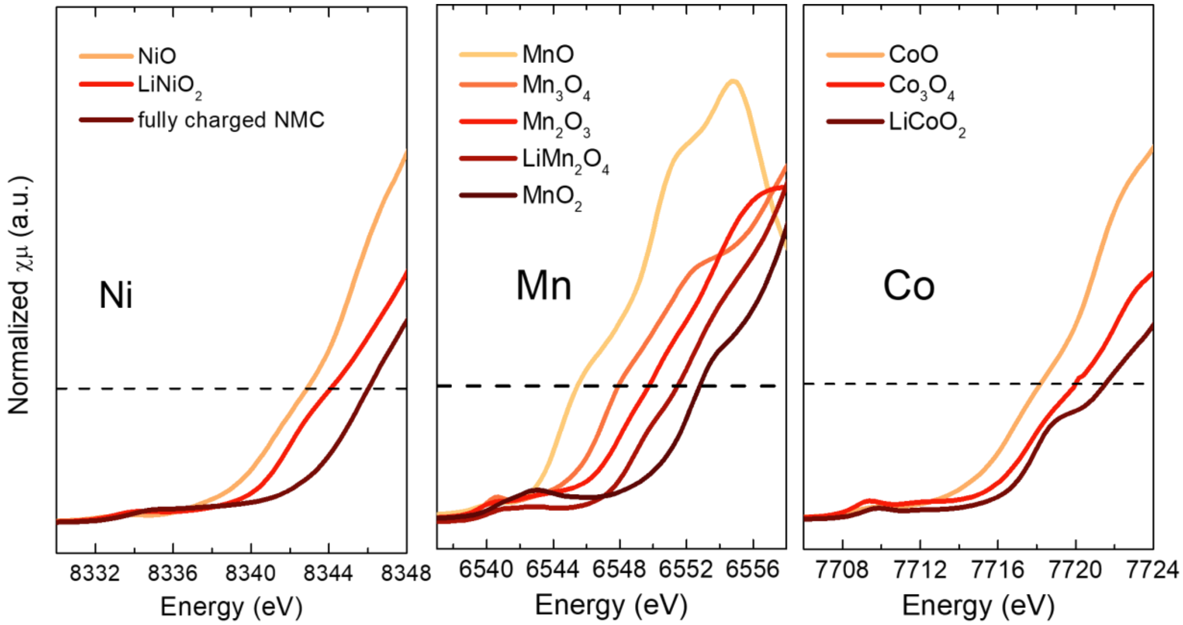

Supplementary Figure 2. The measured X-ray absorption spectra of various transition metal oxides. The dash line corresponds to the half-height of the normalized edge jump (edge position). Fully charged NMC in the left panel refers to $LiNi_{1/3}Mn_{1/3}Co_{1/3}O_2$ charged to 5.3 V to ensure full delithiation.

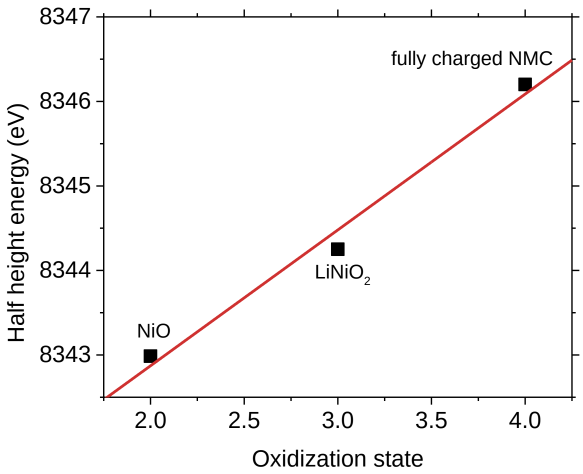

Supplementary Figure 3. The edge positions (defined as half height energy) of various nickel oxides. Fully charged NMC refers to $LiNi_{1/3}Mn_{1/3}Co_{1/3}O_2$ charged to 5.3 V to ensure full delithiation.

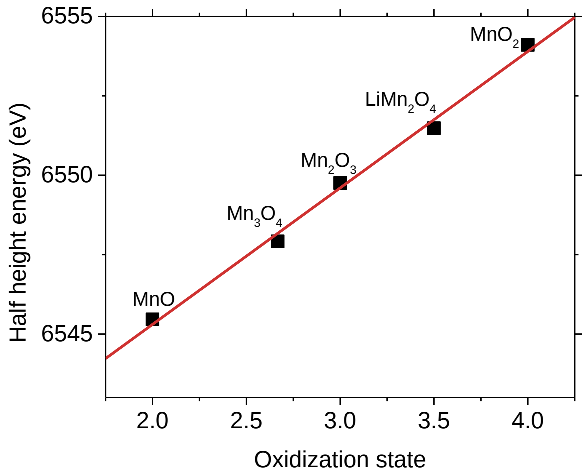

Supplementary Figure 4. The edge positions (defined as half height energy) of various manganese oxides.

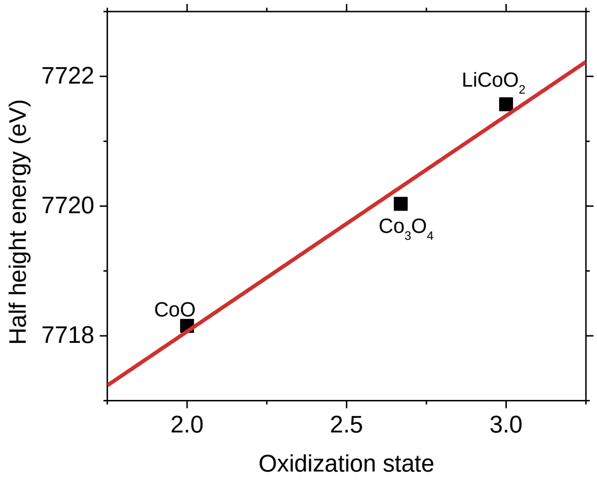

Supplementary Figure 5. The edge positions (defined as half height energy) of various cobalt oxides.

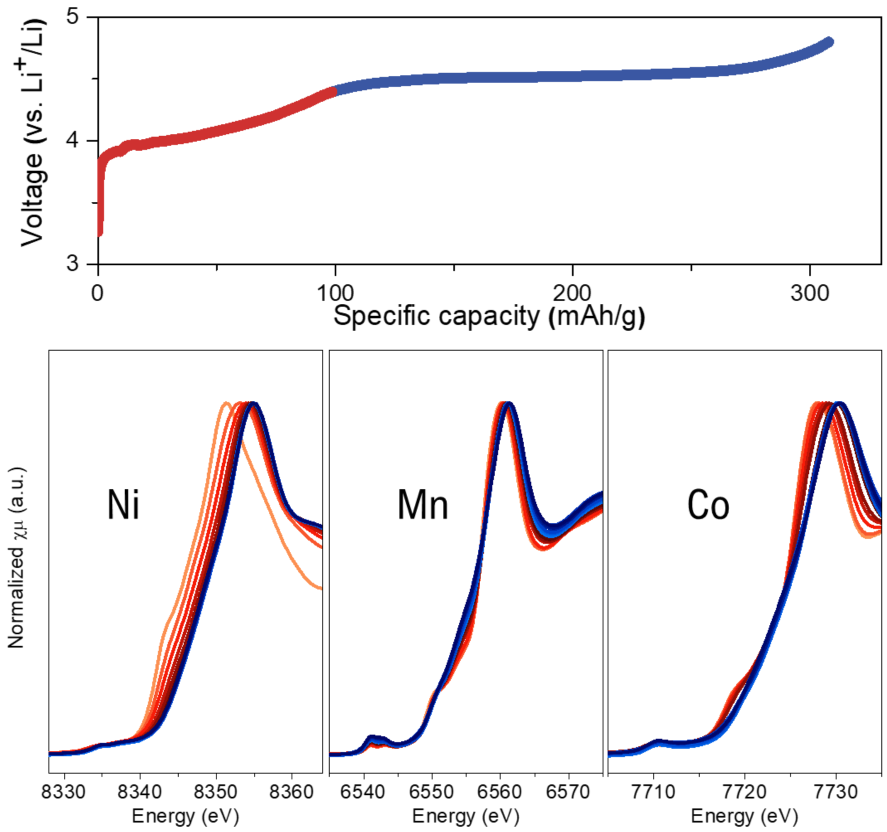

Supplementary Figure 6. First cycle charge profile (upper panel) and in situ XAS of Ni, Mn, and Co during the 1st cycle charge process (lower panel). Orange to red spectra correspond to the charge process before the 4.5 V plateau and the light blue to dark blue spectra correspond to the 4.5 V plateau.

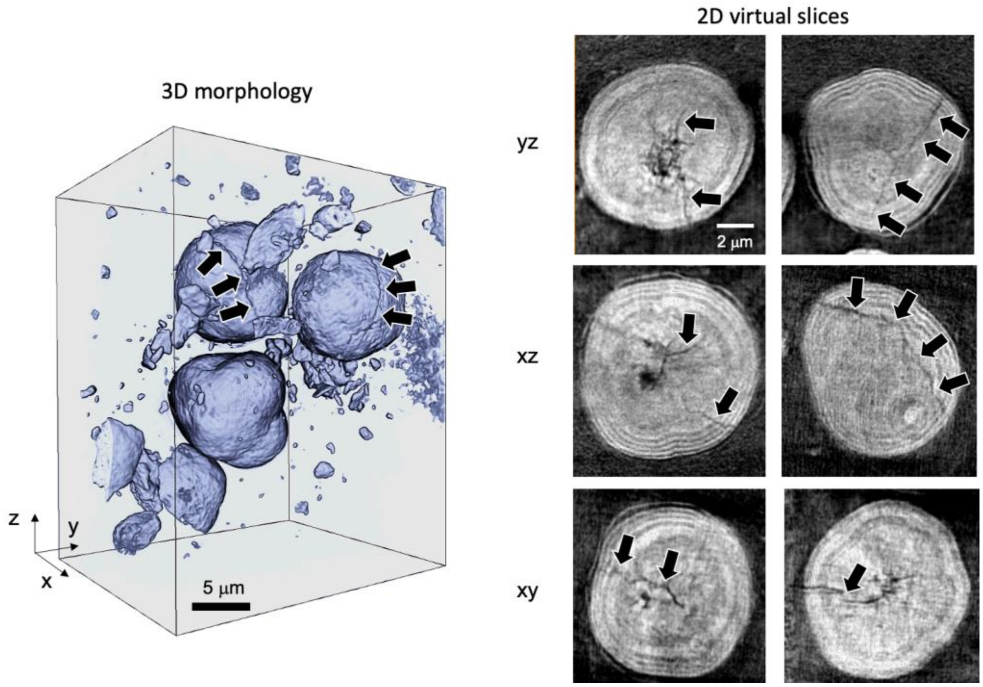

Supplementary Figure 7. Synchrotron nano-tomographic data on Li-rich layered cathode particles that were cycled for 50 times under a rate of 0.5C. Cracks (see back arrows) are observed in both the 3D rendering on the left and selected 2D virtual slices on the right. It worth pointing out that the cracks are not confined to the particles' core regions and propagate through the multiple shell layers.
